# Supplementary material for: Functional Ex Vivo Testing of Alveolar Monocytes in Patients with Pneumonia-Related ARDS
Source: Cells. 2021 Dec 15;10(12):3546. doi: 10.3390/cells10123546 (PMC8700060; doi:10.3390/cells10123546)
Supplement: Supplementary file 1 [file cells-10-03546-s001.zip › cells-1487442-supplementary.pdf]

Online supplement

Functional ex vivo testing of alveolar monocytes in patients with pneumonia-related ARDS

Additional Methods

BAL fluid and blood sampling

BAL fluid was collected from all ARDS patients during a bronchoscopy within 48 hours of ARDS onset. BAL fluid samples were also collected from control patients. Concomitant heparin anticoagulated blood samples were obtained in ARDS and control patients. Samples were stored at room temperature and analyzed within 2 hours.

During a standard flexible bronchoscopy, the bronchoscope was wedged within a bronchopulmonary segment. Four aliquots of normal saline (50 mL each) were instilled through the bronchoscope within the selected bronchopulmonary segment.

After each aliquot was instilled, saline was retrieved using a negative suction pressure. BAL samples were filtered through a 100µm cell strainer; centrifugated and BAL cells were then collected in phosphate buffered saline solution. BAL fluid cytology was performed by direct microscopy after centrifuging broncho-alveolar lavage fluid samples (12 000 revolutions for 10 min) and dying under the May-Grünwald-Giemsa staining. Total (quantified in cells/mL) and differential (i.e., percent of neutrophils, macrophages and lymphocytes) cell counts were measured as recommended [1].

Phagocytosis assay

After treatment with *E. coli* LPS (200 ng/ml) or medium alone, a fraction of blood and BAL fluid samples were incubated 30 min with 20 µL of fluorescent *E. coli* particles (*fluorescent pHrodo™ Green E.coli BioParticles*, Thermo Fisher Scientific, Waltham, MA, USA), as recommended by the furnisher, either at 37°C and 5% CO_2 to evaluate the phagocytic activity of cells or at +4°C to assess fluorescent background for bound but not internalized bacteria. At the end of the incubation period, all samples were put into ice to stop the phagocytosis process. Red cells were then lysed using pHrodo™ BioParticles® Phagocytosis kit for flow cytometry (Thermo Fisher Scientific, Waltham, MA, USA), following the furnisher's instructions. After two successive washings done with 1X Phosphate buffered saline (PBS), cells were further immunophenotyped. Phagocytosis was quantified on both alveolar and circulating monocytes using Fl1/FITC channel and expressed both in % of positive cells and in delta MFI by retrieving basal MFI of unstained cells.

Immunophenotyping

Blood and BAL fluid surface immunostaining was performed as follows: 100 µL of whole blood or BAL fluid were incubated during 15 min at room temperature in the dark with the following conjugated-monoclonal antibodies: CD14-ECD/ HLADR-PACBLUE/ PDL1-BV785 and, for alveolar samples CD45-AF700 and CD169-AF647

were added to identify alveolar monocytes [25] (all antibodies were from BD Biosciences, San Jose, CA, USA), eBioscience (San Diego, CA, USA) or Beckman Coulter (Brea, CA, USA). A *LIVE/DEAD™ Fixable Aqua Dead Cell Stain Kit* (Thermo Fisher Scientific, Waltham, MA, USA) was added to this panel to determine cellular viability. After washing steps, cells were fixed with PBS 1% PFA and further acquired with a LSRII cytometer (BD Biosciences, San Jose, CA, USA) within 48 hours. Flow cytometer analyses were performed with the FlowJo software (version 10 ; *Tree Star*, Ashland, OR, USA). HLA-DR and PD-L1 quantification was expressed in percentages of positive monocytes (% positive cells) among the total monocyte population or in delta MFI. Flow cytometry results were expressed both in percentage of positive cells and delta MFI. A difference might be observed between percentage of positive cells and MFI as these provide different information: percentage of positive cells reflects the amount of monocytes expressing the biomarker of interest, while MFI is the geometric mean of the biomarker fluorescence on each monocyte.

Assessment of TNF production by intracellular staining

After treatment with *E. coli* LPS (200 ng/ml) or medium alone, blood and BAL fluid samples were immunophenotyped and further used to quantify intracellular TNF synthesis according to the regular PerFix-no centrifuge (nc) (Beckman Coulter, Brea, Calif) procedure. In brief, fixation of the samples was conducted for 20 min at room temperature, applying 200 µL of PerFix-nc Fixative Reagent, followed by 20 min permeabilization and concomitant staining at room temperature in the dark. Dried TNF-PC7 antibodies from BD Biosciences (San Jose, CA, USA) and eBioscience (San Diego, CA, USA) were resuspended extemporaneously with 50 µL of the permeabilizing reagent per staining before use. Finally, the reaction was terminated by adding 2.5 mL of PerFix-nc final reagent. Cells were then fixed with PBS 1% PFA and further acquired with a LSRII cytometer (BD Biosciences, San Jose, CA, USA) within 48 hours. Flow cytometer analyses were performed with the FlowJo software (version 10 ; *Tree Star*, Ashland, OR, USA). TNF quantification was expressed in percentages of positive monocytes (% positive cells) among the total monocyte population (positivity threshold was defined with non-stimulated values from the same donor) or in delta MFI.

Figure S1. **Gating strategy of flow cytometric analyses of monocytes in broncho-alveolar lavage fluid (a,b) and blood (c,d).** In broncho-alveolar lavage (BAL) fluid, we represented living singlet cells on a CD14 versus CD45 (the leukocyte common antigen) dot blot. The subsequent CD14+ CD45+ cells were then examined in a forward scatter-area (FSC-A) versus CD169 dot blot to distinguish alveolar monocytes (FSC-AlowCD14+ CD169-) from alveolar macrophages (FSC-AlowCD14+ CD169+) [2]. Then, HLA-DR and PD-L1 expression as well as Phrodo (*E. coli* fluorescent particles) internalization were assessed on alveolar monocytes (panel a). To set up the positive threshold of HLA-DR and PD-L1 staining from background, we considered both the autofluorescence of unstained cells and the unspecific staining detected on CD45- CD14- cells (not shown). For Phrodo/*E. coli* internalization, we considered the autofluorescence of unstained cells and Phrodo background due to its binding on cell surface. Phrodo background was evaluated on alveolar cells incubated at +4°C. The LPS-induced TNF expression was assessed in alveolar monocytes after a step of permeabilization (panel b). To set the positive threshold of TNF staining, we considered the background observed in unstimulated control cells. For blood samples, we represented living singlet cells on a FSC-A versus CD14 dot blot (panel c). Monocytes were then identified as FSC-Alow CD14+ cells. Then, HLA-DR and PD-L1 expression as well as Phrodo internalization were assessed on FSC-Alow CD14+ circulating monocytes. To set up the positive threshold of HLA-DR and PD-L1 staining from background, we considered the autofluorescence of unstained cells and the unspecific staining detected on FSC-Alow SSC-A (side scatter-area) low non-granulocytic and non-myeloid cells (not shown). For Phrodo/*E. coli* internalization, we considered the autofluorescence of unstained cells and Phrodo background due to its binding on cell surface. Phrodo background was evaluated on circulating monocytes incubated at +4°C. The LPS-induced TNF expression was assessed in circulating monocytes after a step of permeabilization (panel d). To set the positive threshold of TNF staining, we considered the background observed in unstimulated control circulating monocytes.

a.

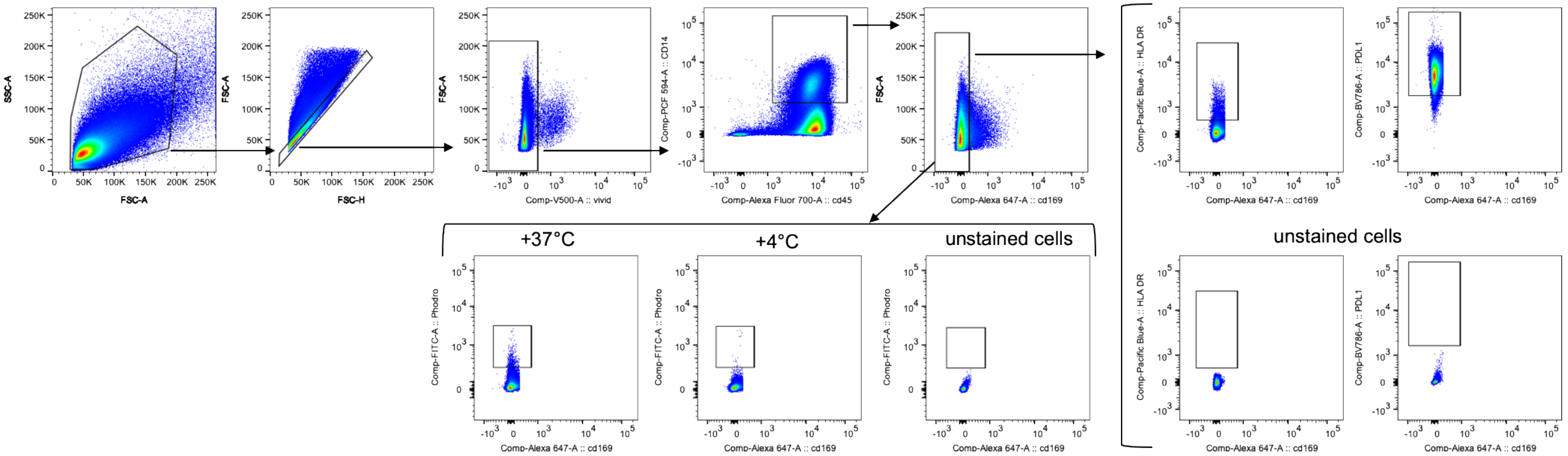

b.

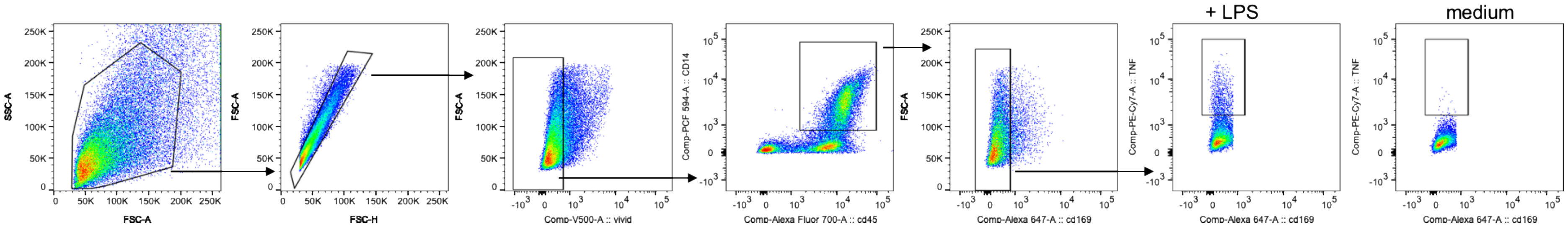
+ LPS
medium
SSC-A
FSC-A
FSC-H
Comp-V500-A :: vivid
Comp-PCF 594-A :: CD14
Comp-Alexa Fluor 700-A :: cd45
Comp-Alexa 647-A :: cd169
Comp-PE-Cy7-A :: TNF

c.

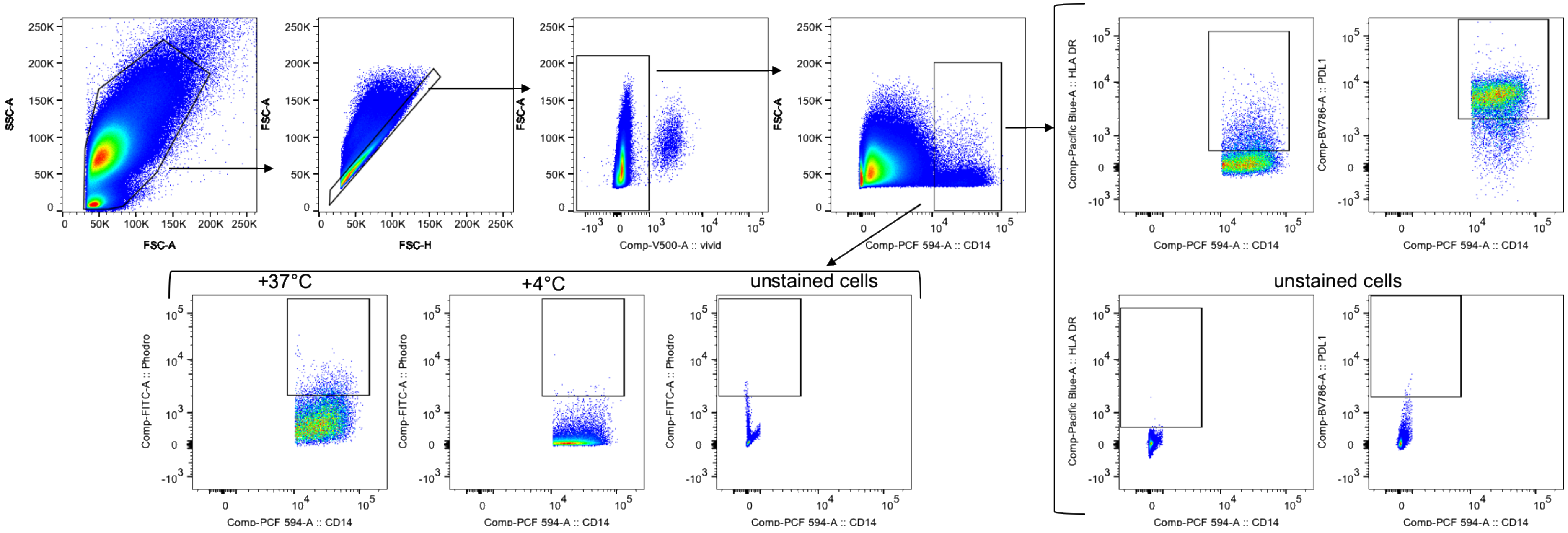
SSC-A
FSC-A
FSC-H
Comp-V500-A :: vivid
Comp-PCF 594-A :: CD14
Comp-Pacific Blue-A :: HLA DR
Comp-BV786-A :: PDL1
+37°C
+4°C
unstained cells
Comp-FITC-A :: Phrodo
unstained cells

d.

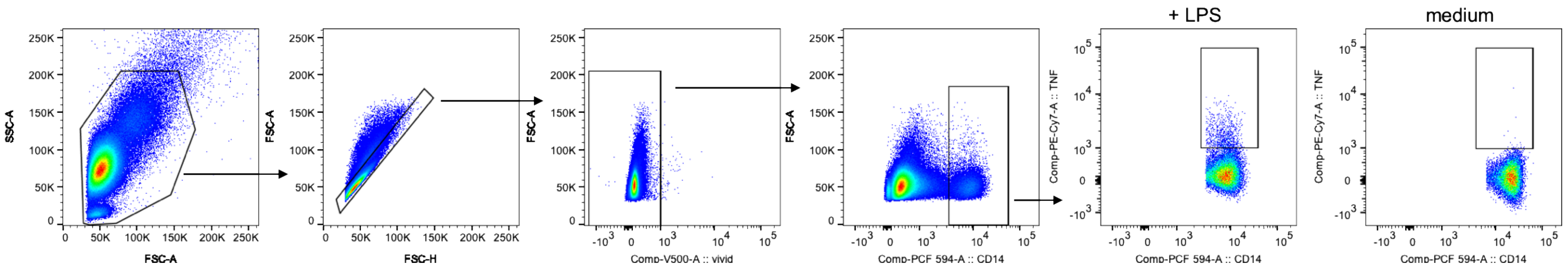

+ LPS
medium
SSC-A
FSC-A
FSC-H
Comp-V500-A :: vivid
Comp-PCF 594-A :: CD14
Comp-PE-Cy7-A :: TNF

Figure S2. **Phagocytosis of *E. coli* particles by alveolar monocytes according to their HLA-DR expression measured by flow cytometry.** The phagocytosis activity of alveolar monocytes has been measured with and without (medium condition) LPS challenge, and expressed in percentage of positive cells (%, a) and in mean fluorescence intensity (MFI, b), in ten pneumonia-related ARDS patients. Phagocytosis activity was analyzed by two-way ANOVA with repeated measures. When results were expressed in % (a), there was no significant effect of HLA-DR status (HLA-DR$^-$ vs HLA-DR$^+$, p=0.2687), nor a significant effect of experimental condition (medium vs LPS, p=0.1228), or a significant interaction (HLA-DR status x experimental condition, p=0.7057). When results were expressed in MFI (b), there was no significant effect of HLA-DR status (HLA-DR$^-$ vs HLA-DR$^+$, p=0.0621), nor a significant effect of experimental condition (medium vs LPS, p=0.8041), or a significant interaction (HLA-DR status x experimental condition, p = 0.9984). Displayed p-values come from post-hoc comparisons performed using the Sidak test.

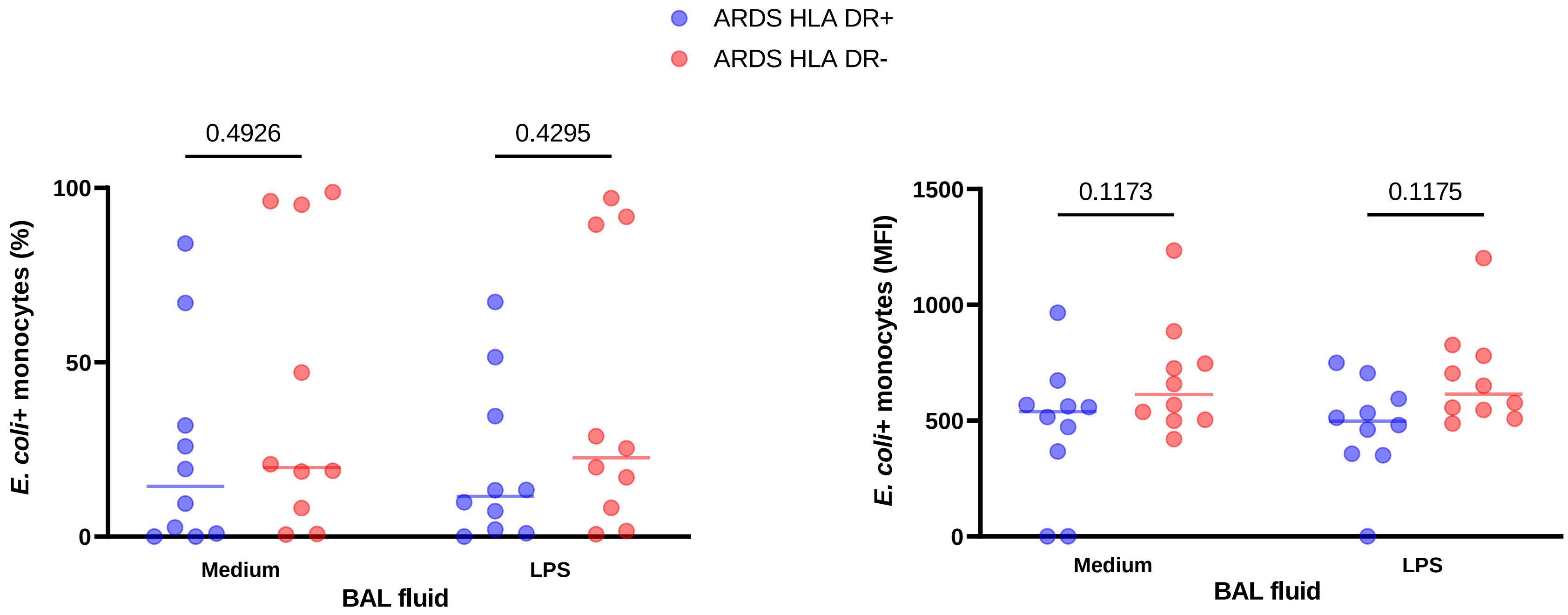

Figure S3. **Alveolar monocytes intracellular TNF expression according to HLA-DR expression measured by flow cytometry.** The intracellular TNF expression of alveolar monocytes was measured with and without (medium condition) LPS challenge and expressed in percentage of positive cells (%, a) and in mean fluorescence intensity (MFI, b), in ten pneumonia-related ARDS. By two-way ANOVA with repeated measures, when results were expressed in % (a), there was no significant effect of HLA-DR status (HLA-DR^- vs HLA-DR^+, p=0.9500), but a significant effect of experimental condition (medium vs LPS, p=0.0029), and no significant interaction (HLA-DR status x condition, p=0.7458). When results were expressed in MFI (b), there was no significant effect of HLA-DR status (HLA-DR^- vs HLA-DR^+, p=0.8459) but a significant effect of experimental condition (medium vs LPS, p=0.0076), and no significant interaction (HLA-DR status x experimental condition, p = 0.3994). Displayed p-values come from post-hoc comparisons performed using the Sidak's test. Bolded p values are significant at the <0.05 level. Horizontal bars represent median values.

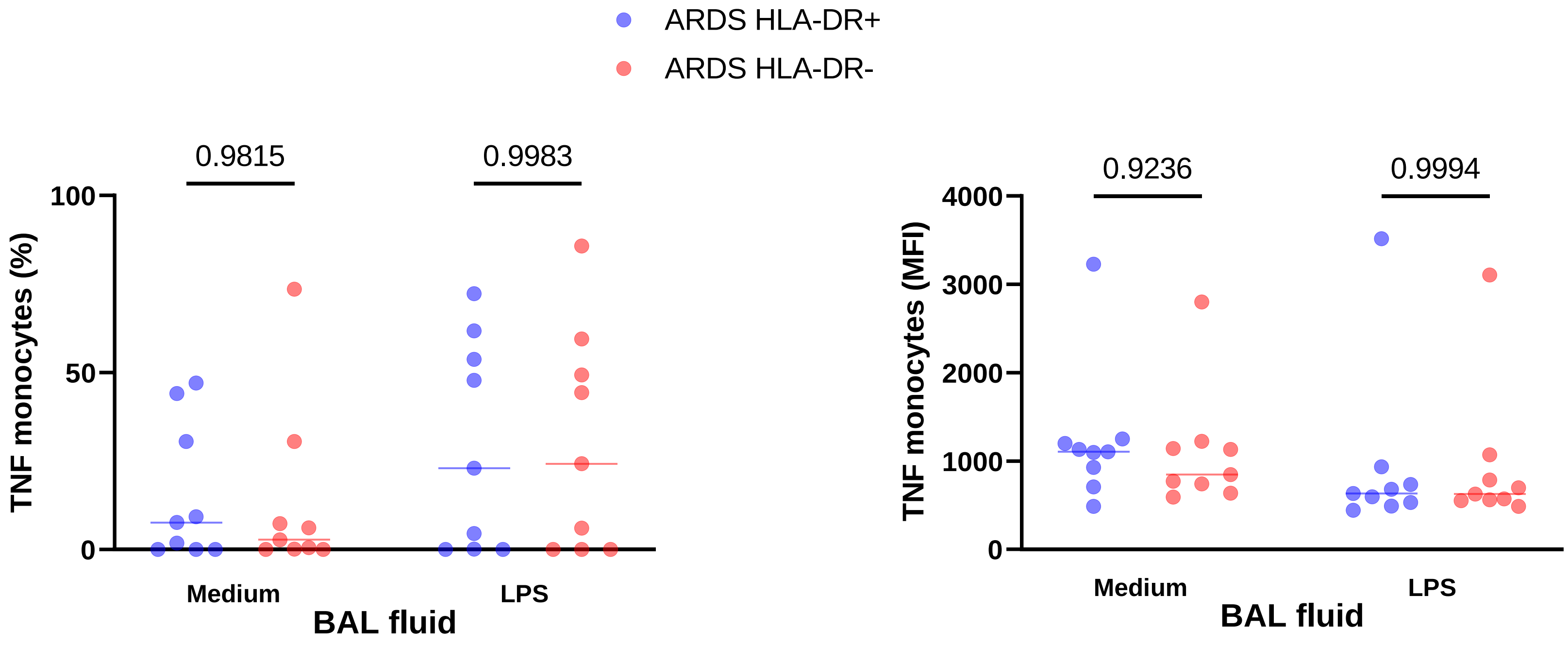

Table S1. Characteristics of non ARDS patients (n=7) included in the study

**Variables**	**Non-ARDS patients**[a]						
	1	2	3	4	5	6	7
**Age**	40	72	30	25	40	25	67
**Gender**	Male	Male	Male	Male	Female	Male	Male
**Immunosuppression**	No	No	No	No	No	No	No
**Comorbidities**	None	Prostate cancer (2005, in remission)	None	None	None	None	Ischemic heart disease, hypertension, COPD
**Smoker**	No	Yes	Yes	No	No	No	Yes
**Clinical indication for bronchoscopy**	Micronodules in RUL	Ground glass lesions in right upper lobe + nodular lesion	Suspicion of tuberculosis	Hilar adenopathy	Lung micronodules and positive IFN-γ release assay	Weight loss, hilar bilateral lymphadenopathy and interstitial abnormalities	Pulmonary isolated cysts in RLL in context of medical history of cerebral adenocarcinoma and smoking
**Characteristics upon BAL sampling**							
**WBC counts, 10^3/mm^3**	6.4	17.4	5.0	6.0	6.4	6	12.2
Neutrophils	3.5	14.4	2.4	3.7	3.5	3.7	9
Lymphocytes	2.4	2.2	1.5	1.3	2.4	1.3	1.3
Monocytes	0.4	0.3	0.6	0.8	0.4	0.7	0.7

**BAL fluid cytology**							
Leukocytes, 10^3/mL	77	70	197	130	77	130	143.3
Macrophages, %	87	84	66	66	87	66	29
Neutrophils, %	1	15	1	1	0	0.5	1
Lymphocytes, %	12	1	30	33	13	33	49

[a]All non ARDS patients were free of ARDS, active pulmonary infection, infiltrative lung disease, and immunosuppression; BAL, broncho-alveolar lavage; RLL, right lower lobe; RUL, right upper lobe; WBC, white blood cells

Table S2. Microbiological documentation of patients (n=10) with pneumonia-related ARDS

Bacteria	**7 (70%)**
Streptococcus pneumoniae	2
Staphylococcus aureus	2
Haemophilus influenzae	1*
Enterobacteriaceae	3
Escherichia.coli	2*
Klebsiella pneumoiae	1
Pseudomonas aeroginosae	1
Virus	**1 (10%)**
Influenza A or B	0
Adenovirus	1
No documentation	**2 (20%)**

* Including three patients with bacterial co-infections

References

1. Meyer, K.C.; Raghu, G.; Baughman, R.P.; Brown, K.K.; Costabel, U.; du Bois, R.M.; Drent, M.; Haslam, P.L.; Kim, D.S.; Nagai, S.; et al. An Official American Thoracic Society Clinical Practice Guideline: The Clinical Utility of Bronchoalveolar Lavage Cellular Analysis in Interstitial Lung Disease. *American Journal of Respiratory and Critical Care Medicine* **2012**, *185*, 1004–1014, doi:10.1164/rccm.201202-0320ST.

2. Yu, Y.-R.A.; Hotten, D.F.; Malakhau, Y.; Volker, E.; Ghio, A.J.; Noble, P.W.; Kraft, M.; Hollingsworth, J.W.; Gunn, M.D.; Tighe, R.M. Flow Cytometric Analysis of Myeloid Cells in Human Blood, Bronchoalveolar Lavage, and Lung Tissues. *Am. J. Respir. Cell Mol. Biol.* **2016**, *54*, 13–24, doi:10.1165/rcmb.2015-0146OC.
